# Epidemiology and antibiogram of common mastitis-causing bacteria in Beetal goats

**DOI:** 10.14202/vetworld.2020.2596-2607

**Published:** 2020-12-08

**Authors:** Abdul Jabbar, Muhammad Hassan Saleem, Muhammad Zahid Iqbal, Muhammad Qasim, Muhammad Ashraf, Mahmoud M. Tolba, Hebatallah Ahmed Nasser, Hira Sajjad, Ayesha Hassan, Muhammad Imran, Imtiaz Ahmad

**Affiliations:** 1 Department of Clinical Medicine, University of Veterinary and Animal Sciences, Lahore, Punjab, Pakistan; 2 Department of Economics, Finance, and Statistics Jonkoping University, Sweden; 3 Department of Theriogenology, University of Veterinary and Animal Sciences, Lahore, Pakistan; 4 Biomedical Informatics and Biotechnology Group, Department of Informatics and Systems, Division of Engineering research, National Research Centre, Cairo, Egypt; 5 Department of Microbiology and Public Health, Faculty of Pharmacy, Helipolis University, Cairo, Egypt; 6 Department of Food Science and Human Nutrition, University of Veterinary and Animal Sciences, Lahore, Punjab, Pakistan; 7 Department of Surgery and Pet sciences, University of Veterinary and Animal Sciences, Lahore, Punjab, Pakistan; 8 Institute of Biochemistry and Biotechnology, University of Veterinary and Animal Sciences, Lahore, Punjab, Pakistan; 9 Department of Veterinary Clinical Sciences, University of Poonch Rawalakot, Azad Jammu and Kashmir, Pakistan

**Keywords:** antibiotic disks, Beetal goats, common bacteria, epidemiology, isolates, mastitis, Pattoki

## Abstract

**Background and Aim:**

Mastitis has been identified as the most prevalent and economically imperative disease among dairy animals. Thus, understanding its common bacterial pathogens and risk factors is necessary to improve udder health at herd, region, or country level. However, scientific research on caprine mastitis, especially on Beetal breed, has remained to be insufficient in Pakistan. Therefore, this study aimed to evaluate the epidemiology and antibiogram assay of common mastitis-causing bacterial agents, that is, *Staphylococcus*, *Streptococcus*, and *Escherichia coli*, in dairy goats.

**Materials and Methods:**

In total, 500 Beetal goats, irrespective of age and those that were not treated with any kind of antimicrobial agents during the past 120 h, were screened using California Mastitis Test in Pattoki, Kasur District, whereas epidemiological factors were recorded. The milk samples of mastitic goats were then collected and processed using standard methods. Each sample was primarily cultured on nutrient agar. Using a specific medium, each bacterial colony was separated using several streak methods. Six antibiotic disks belonging to different antibiotic groups were used for antibiogram profiling of bacterial isolates. Chi-square test was used to assess the association of baseline characteristics and mastitis occurrence. Meanwhile, multivariable logistic regression (p<0.001) was utilized to determine the risk factors associated with positive and negative dichotomous outcome of mastitis.

**Results:**

The results revealed that the overall prevalence of goat mastitis was 309 (61.8%), in which 260 (52%) and 49 (9.8%) cases were positive for subclinical mastitis (SCM) and clinical mastitis (CM), respectively. *Streptococcus* and *E. coli* were found to be the predominant isolates causing SCM and CM, respectively (p<0.001). It was observed that amoxicillin+clavulanic acid was highly sensitive to isolates of *Staphylococcus* and *Streptococcus* and ceftiofur sodium to isolates of *Streptococcus* and *E. coli*., while enrofloxacin was found to be sensitive to isolates of *Streptococcus* and *E. coli*. Risk factors such as herd structure, deworming, vaccination, presence of ticks, use of teat dip and mineral supplements, feeding type, age, parity, housing, blood in the milk, milk leakage, milk taste, and milk yield were found to have the strongest association with mastitis occurrence, while ease of milking has moderate association.

**Conclusion:**

In the area examined, cases of SCM were found to be higher compared with that of CM, and ceftiofur sodium has been identified as the preferred treatment in both clinical and subclinical forms of caprine mastitis in Beetal goats. Risk factors for mastitis that was identified in this study can form the basis for the creation of an udder health control program specific for dairy goats. We hope our findings could raise awareness of the risk factors and treatment approaches for common mastitis-causing bacterial agents.

## Introduction

Worldwide, goats are primarily raised for milk, meat, and fiber production. Across the globe, Pakistan ranks third among countries with the largest goat production, following after India and China. Goats are known to be the poor man’s cow in Pakistan [1-5]. This could be attributed to goat’s milk being cheap, nutritious, easily digestible, and wholesome, which is recommended not only for its nutritional benefits for growing babies but also for its therapeutic effects in many diseases [5-7]. Mastitis has been identified as one of the expensive and multifactorial diseases affecting the udder tissue of dairy animals as this will often result in the decrease in milk quality, quantity, and yield, which in turn leads to drop in the overall milk production and also cause physical, chemical, pathological, and microbiological changes in the milk composition and impaired quality, an increase in somatic cells, especially leukocytes detrimental changes, transitory to permanent blocking of milk ducts, and also having the danger for the spread of milk-related zoonotic diseases [8]. In general, mastitis can either be clinical or subclinical. Clinical mastitis (CM) is often characterized by visible symptoms such as the udder becoming swollen or hot to the touch and changes in the organoleptic properties of milk. Meanwhile, subclinical mastitis (SCM) requires somatic cell count and bacteriological culturing of milk for accurate diagnosis; thus, various tests such as Surf Field Mastitis Test and California Mastitis Test (CMT) should be performed under field conditions [9]. Bacterial pathogens, such as *Staphylococcus* spp., *Streptococcus* spp., *Escherichia coli*, and *Pseudomonas aeruginosa*, have been reported as the main causative agents of mastitis in goats [10-12]. To improve udder health, it is necessary to determine the factors associated with the increased risk of SCM [13]. Reoccurrence of infection or the risk of infection can be attributed to factors such as poor treatment protocol during late lactation [14,15], low body score, long teats, milk fever, season, and prophylactic hygiene management; furthermore, infections in health-care centers have been identified as the major contributors to mastitis prevalence throughout the world [16-18].

Today, mastitis has been determined as the most common and expensive disease that plagued the entire dairy industry, with about two-thirds of its total economic losses attributed to SCM infection. Cases of mastitis in countries like Pakistan have seen an increasing trend due to poor disease prevention and reporting system [19]. Thus, understanding its common bacterial pathogens and risk factors is necessary to improve udder health at herd, region, or country level. In Pakistan, caprine mastitis has not been given enough attention as considerable efforts and resources have been focused on the control of mastitis infection among cattle and buffaloes; furthermore, overall scientific research data on caprine mastitis, especially on the recently documented strains of Beetal goat, are meager or non-existent at all [20-23].

Thus, there is a dire need to curtail this disease to produce milk according to the standards set by the World Trade Organization. This study has been conducted to raise awareness of the prevalence of caprine mastitis, its associated risk factors, and bacterial pathogens such as *Staphylococcus*, *Streptococcus*, and *E. coli* in lactating dairy goats in Pattoki Tehsil, Kasur District, Punjab, Pakistan. The findings of this study will be helpful in the creation of udder health control program specific for dairy goats.

## Materials and Methods

**Figure-1:**
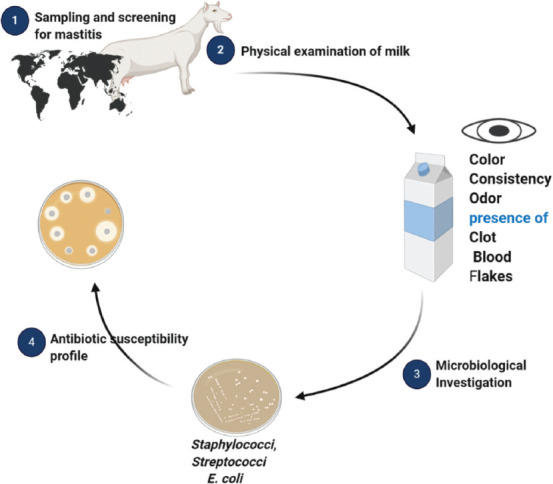
The flowchart of materials and methods.

The flowchart for the Materials and Methods is provided in Figure-1.

### Ethical approval and Informed consent

Ethical approval for this study was obtained from University of Veterinary and Animal Sciences (UVAS), Lahore, Pakistan.

### Study period and location

The study was conducted from July 2019 to December 2019 on both public and private farms and small households of every Union Council of Pattoki Tehsil, Kasur District, Punjab, Pakistan.

### Sample size

The sample size was calculated based on the expected prevalence of about 20% (known disease status), with confidence intervals (CI) of 95% and absolute desire precision 5%. The expected prevalence of 20% was deduced from the average prevalence of caprine mastitis in the previous studies reported from 2013 to 2018 (6 years) [5,22,24,25].

The sample size was then estimated using the following equation [26]:

n = 1.96^2^ P_exp_ (1−P_exp_)/d2

d = 1- C.I where:

n = required sample size

Pexp = expected prevalence

d = desired absolute precision.

The number of samples thus calculated was further subjected to the following equation for adjustments to reach the maximum number of samples [26]:

nadj = (N × n)/(N+n) where:

N = total population

n = calculated sample size through formula

Following the proportionate sampling strategy and assuming the highest population (70%) of Beetal goats in Punjab (5), 500 Beetal goats were screened for this study, examining the prevalence of mastitis in Pattoki Tehsil, Kasur District.

### Sampling and screening for mastitis

**Figure-2:**
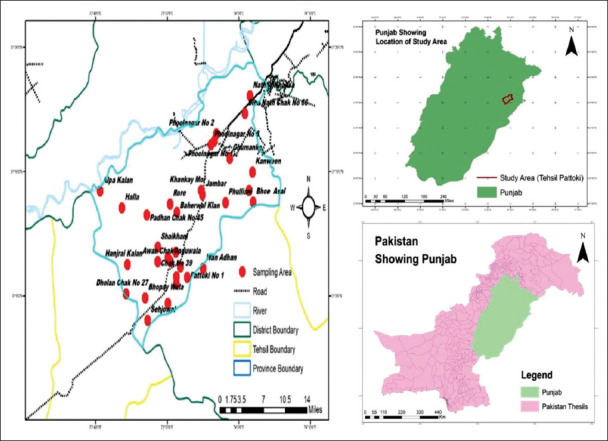
Sampling and screening for mastitis, study area, were generated with the help of ARC GIS version 10.3.

In total, 500 goats, irrespective of age, which were not treated with any kind of antimicrobial agents in the past 120 h, were screened using CMT as per the procedure described by Saleem [4], from July 2019 to December 2019. The study was conducted on both public and private farms and small households of every Union Council of Pattoki Tehsil, Kasur District, Punjab, Pakistan. Coordinates of sample unit were taken using GPS Essential Android application, while the study spots were generated using ArcGIS version 10.3 (Figure-2). Ten milliliters of CMT-positive milk samples were collected from each animal using sterile plastic tubes with safety lids, in compliance with the National Mastitis Council (USA) procedures [27]; these were later stored in the isothermal container at 2-4°C and were immediately transported to the University Diagnostics Laboratory, Central Laboratory Complex (CLC) of UVAS Ravi Campus, Pattoki, for further analysis.

### Physical examination of milk

Immediately after collection, the milk samples were subjected to naked eye examination to determine any changes in color, consistency, or odor, any presence of clot, blood or flakes, and other visible abnormalities.

### Risk factor analysis

Data on the potential risk factors such as herd structure, deworming, vaccination against common diseases, presence of ticks, use of teat dip and mineral supplements, feeding type, age, parity, housing, blood in the milk, milk leakage, milk taste, milk yield, and ease of milk were obtained using the questionnaire for each sampling unit, assuming that these risk factors are determinants of the disease. Udder tick infestation was considered when more than 2 ticks were present as well as the presence of tick on teat was including ticks portion as per described by Abera *et al*. [28].

### Microbiological investigation

**Figure-3:**
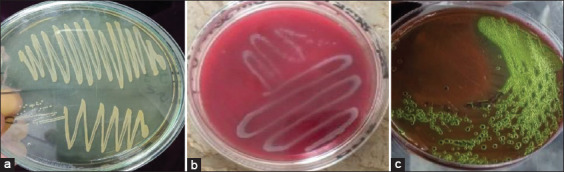
Isolated of (a) staphylococci on staph-110 agar, (b) streptococci on blood agar, and (c) *Escherichia coli* on eosin methylene blue agar.

Milk samples were cultured using a conventional technique as described by Khan *et al*. [29]. Plates were then incubated for 24 h at 37°C, where the growth of bacteria was observed after every colony was separated using the specific media as follows: Staph-110 for *Staphylococcus*, blood agar for *Streptococcus*, and eosin methylene blue agar for *E. coli*. Separate colonies were purified using several streaking methods. Pure bacterial isolates were determined using their baseline cultural characteristics; guidance for the microscopic study and biochemical profile was taken from the identification flowcharts of Bergey’s Manual of Systematic Bacteriology (2010). Isolates of *Staphylococcus* were confirmed using the procedure described by Nazia *et al*. [30] while the method of Gillespie and Oliver [31] was used for the confirmation of streptococci and *E. coli* isolates. Different isolates found on specific media are shown in Figure-3a-c.

### Prevalence of pathogen

The prevalence of mastitis-causing bacterial pathogens was established by reviewing epidemiological surveys and treatment trials published from 1995 to 2018. The positive milk samples were cultured for analysis, and the prevalence was calculated as per the formula described by Waltner-Toews [26].


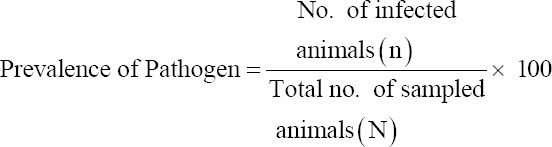


### Antibiogram profile

For the antibiogram profiling of all bacterial isolates, antibiotic disks of enrofloxacin (ENO) (5 µg), amoxicillin+clavulanic acid (20/10 μg), oxytetracycline (30 μg), ceftiofur sodium (30 μg), gentamycin (10 μg), and sulfamethoxazole+trimethoprim (1.25/23.7 μg) were exposed using the disk diffusion method to test for antibiotic sensitivity [32]. The inhibition zones (mm) that have formed around disks were measured after incubation at 37°C for 24 h. The standards set by the Clinical and Laboratory Standards Institute (2020) was used to compare the inhibition zones of each disk, to determine sensitive, intermediate, or resistant susceptibility.

### Statistical analysis

The collected data set was then encoded into SPSS (IBM Corp., NY, USA) version 26.0. Frequencies of baseline characteristics were reported. To determine the risk factors associated with mastitis occurrence in goats, all variables were initially tested through univariable analysis [33,34]. The Chi-square (χ^2^) test was used to assess the association of baseline characteristics and the occurrence of mastitis. Multivariable logistic regression (MLR) was also used to recognize risk factors associated with positive and negative dichotomous outcome of mastitis. MLR-based statistical test was based on two-sided Wald test, and 95% CI was used to highlight the odds ratio. The statistical significance was set for all statistical tests at p<0.05 (two-sided).

## Results and Discussion

### Baseline characteristics of the study

The baseline characteristics used in this study were selected randomly in a blinded manner. After sampling, it was observed, animals having age of 2-3 and 3-4 years were 19% in each group, 46.8% parity ≥5 times, and 78% history of fodder feeding in the studied animal, as shown in Table-1.

**Table-1:** Baseline characteristics of the study.

Characteristics	*n	%
Age (years)		
2-3	95	19
3-4	95	19
4-5	72	14.4
5-6	67	13.4
6-7	64	12.8
7-8	56	11.2
≥9	51	10.2
Parity		
1-2	129	25.8
3-4	137	27.8
≥5	234	46.8
Feeding		
Fodder	390	78
Fodder+Concentrate	51	10.2
Concentrate	59	11.8

* n=500 respondents

### Epidemiology of Beetal goat mastitis in Pattoki Tehsil

Goat mastitis has not been given enough attention in Pakistan [20,21]. Thus, our analysis has revealed that the overall prevalence of mastitis was 309 (61.8%), in which 260 (52%) and 49 (9.8%) were positive for SCM and CM, respectively, whereas 191 (38.2%) were found to be negative in the study area (Table-2). These findings are consistent with that of Sori *et al*. [35] and Matios *et al.*, [36], who reported that the prevalence range of mastitis is about 52.8-64.4%. In Peshawar, the mastitis incidence rate in lactating goats has been reported to be 80.0%, 26.7%, 33.3%, 57.1%, and 42.0%, respectively, for five successive years (1997-2001) [37]. It is reported that the overall prevalence of goat mastitis is 40.4% [38], having an average of 18.3% occurrence rate among different goat breeds in Pakistan [22]. It is reported that the prevalence range of goat mastitis is 7-40%, 5-30%, and 20-50%, respectively [39-41], while 33%, 30.6%, 44.6%, 36%, 33.9%, and >50% prevalence are, respectively, reported by Aqib *et al*. [15], Ali *et al*. [21], Hall and Rycroft [42], Islam *et al*. [43], and Bourabah *et al*. [44]. This variation could be due to environmental factors and varying animal breed, fluctuation of immune response, housing and management system, all other farmers’ and host’s-related determinants, usage of different diagnostic methods and different levels of expertness for diagnosis, and the varying interpretation of the results [45].

**Table-2:** Prevalence of bacterial species in SCM and CM in Beetal goats.

Bacterial isolate†	CM (9.8%)	SCM (61.8%)
Staphylococci	46.9	15
Streptococci	14.3	86.5*
*Escherichia coli*	91.8*	48.5

† There were 309 positive samples out of 500 (total samples). 260/500 SCM and 49/500 CM. SCM=Subclinical mastitis, CM=Clinical mastitis

Our findings of SCM are close to that of Najeeb *et al*. [24], Oliveira *et al.*, [46], who have reported that the prevalence rate of SCM in goats using CMT in Thika East Sub-county, Kenya, was 53% and 50.9%, respectively. Similarly, our findings of CM are close to that of Ferdous *et al*. [47], who reported 11.67% and 8% prevalence rate for CM in Bangladesh [38]. Oppositely to our findings, the prevalence of SCM is 76.7% and 15-79%, respectively, in goats, when screened with CMT [48,49]. Similarly, prevalence SCM is 32.4%, 39.2%, 38%, 40.2%, 47%, 14.3%, 38.8%, 19.9%, and 35.5%, respectively, by Saleem [4], Ali *et al*. [21], Altaf *et al*. [33], Danmallam and Pimenov [38], Moroni *et al*. [47], Hussain *et al*. [50], Ferdous *et al*. [51], Mishra *et al*. [52], and McDougall *et al*. [53], while opposite to the study prevalence finding of CM, which is 3.6% [54]. This prevalence can vary depending on the place and its conditions.

### Prevalence of pathogens

Among goats, bacterial pathogens such as *Staphylococcus* spp., *Streptococcus* spp., and *E. coli* were reported as the main causative organisms of mastitis [12]. As it was studied that SCM in goats is mainly of bacterial origin, these three species of bacteria from milk samples positive for mastitis were isolated [41]. The percentages of *Staphylococcus*, *Streptococcus*, and *E. coli* in CMT-positive samples were 46.9%, 14.3%, and 91.8%, respectively, for CM and 15.0%, 86.5%, and 48.5%, respectively, for SCM, as shown in Table-2. It was further revealed that *Streptococcus* was predominant in SCM and *E. coli* in CM as p<0.005. These findings are close to that of Dieser *et al*. [55], who reported that in SCM, *Streptococcus* prevalence was 57.3%; meanwhile, Bradley and Green [56] reported *E. coli* as the predominant pathogen isolated on all farms in all months of the year, which is opposite to our findings. On the other hand, Hussain *et al*. [51] reported *Staphylococcus* as the most common pathogen (51.4%) isolated. The results regarding isolated organisms were consistent with the results reported as *Staphylococcus aureus* (45.3%), *Streptococcus* spp. (22.7%), *E. coli* (11.6%), and *Klebsiella* spp. (3.7%) [21]. Similar findings were also reported by Islam *et al*. [44], with 20.8-46.6% prevalence of *S. aureus* as a pathogen of SCM. In another study, it was concluded that most SC intramammary infections were caused by coagulase-negative *Staphylococcus* spp. [21]. It was reported that *Staphylococcus* is more dominant in Pakistan in terms of SCM infections [4]. Similarly, it was reported that *S. aureus* was predominant in milk samples [21], while Manser (2000) recorded the incidence of catalase-negative *Staphylococcus* and catalase-positive staphylococci at 80% and 16%, respectively. It was also reported that 38.98%, 27.1%, and 10.2% of *Staphylococcus* spp., *E. coli*, and *Bacillus* spp., respectively, were found to be in goats positive for SCM, which was opposite to our results [49]. It was further reported that staphylococci were the major etiological agents of SCM in Bangladesh and Pakistan [21,57], thus identifying staphylococci as the predominant isolate for SCM cases [58]. In total, 38.98%, 27.1%, and 10.2% of *Staphylococcus* spp., *E. coli*, and *Bacillus* spp., respectively, were isolated from goats positive for SCM [49].

As *E. coli* is mostly found on farm animals, it has been recognized as the most common environmental contaminant [59,60]. Our findings are very close to that of Moroni *et al*. [49], who reported *E. coli* as the second most common pathogen found in milk samples positive for SCM. Meanwhile, *Staphylococcus* was also found to be the highest in Kenya (60.3%), Spain (70%), and the USA (38.2%) [61-63]. These differences could be attributed to management conditions or geographical locations of the farm [64].

### Antibiotic susceptibility profile

It was observed that amoxicillin+clavulanic acid was highly sensitive to 58/62 isolates of *Staphylococcus* (p<0.001), with mean±standard deviation (SD) (18.3±0.2), and 221/232 isolates of *Streptococcus* with mean±SD (20.2±1.3). Similarly, ceftiofur sodium was determined to be highly sensitive to 221/232 isolates of *Streptococcus*, with mean±SD (22.3±0.7), and 162/171 isolates of *E. coli* with mean±SD (21.3±0.02). Meanwhile, ENO was found to be sensitive to 217/232 isolates of *Streptococcus*, with mean±SD (25±2.5), and 138/171 isolates of *E. coli* with mean±SD (22.3±0.8; p<0.01). The sensitivity zones of the antibiotic disks to bacterial isolates are shown in Table-3, whereas the prevalence and antibiotic susceptibility of the isolated *Staphylococcus*, streptococci, and *E. coli* are shown in Table-4.

**Table-3:** Sensitivity zone (mean±SD) in mm of antibiotic disk against bacterial isolates.

Antibiotic disk	Staphylococci	Streptococci	*Escherichia coli*
		
	Mean±SD	Mean±SD	Mean±SD
ENO-5	21.4±0.2	25±2.5	22.3±0.8
AMC-5	18.3±0.2	20.2±1.3	20.4±1.3
T-30	8.9±7.3	28±1.3	26.7±0.5
XNL-30	22±0.7	22.3±0.7	21.3±0.2
GM-10	16.1±0.8	16.4±0.9	17±1.3
SXT	17.8±0.6	17.05±0.7	17.3±0.2

Six different antibiotics are used to determine the sensitivity zone of bacteria. ENO-5=Enrofloxacin (5 μg), AMC-5=Amoxicillin+Clavulanic acid (20/10 μg), T-30=Oxytetracycline (30 μg), XNL-30=Ceftiofur sodium (30 μg), GM-10=Gentamycin (10 μg), and SXT=Sulfamethoxazole trimethoprim (1.25/23.7 μg). Data are presented as mean±SD.

**Table-4:** Prevalence and antibiotic susceptibility of the isolated *staphylococci, streptococci*, and *Escherichia coli*.

Antibiotic disks	Staphylococci (62 a)			Streptococci (232 b))			*Escherichia coli* (171 c))		
		
	S (%)	I (%)	R (%)	S (%)	I (%)	R (%)	S (%)	I (%)	R (%)
ENO-5	5 (8.1)	2 (3.2)	55 (88.5)	217 (93.5)*	10 (4.3)	5 (2.2)	138* (80.7)	21 (12.3)	12 (7)
AMC-5	58 (93.5)**	3 (4.8)	1 (1.6)	221 (95.3)**	8 (3.4)	3 (1.3)	2 (1.2)	0 (0.0)	169 (98.8)
T-30	3 (4.8)	7 (11.3)	52 (83.9)	28 (12.1)	27 (11.6)	177 (76.3)	3 (1.8)	7 (4.1)	161 (94.2)
XNL-30	11 (17.7)	10 (16.1)	41 (66.1)	221 (95.3)**	10 (4.3)	1 (0.4)	162 (94.7)**	6 (3.5)	3 (1.8)
GM-10	4 (6.5)	13 (21)	45 (72.6)	25 (10.8)	54 (23.3)	153 (65.9)	4 (2.3)	8 (4.7)	159 (93)
SXT	2 (3.2)	9 (14.5)	51 (82.3)	2 (0.9)	56 (24.1)	174 (75)	24 (14)	24 (14)	123 (71.9)

There were 39 and 23, 225 and 7, 126 and 45;

a, b and C in SCM and CM, respectively;

** highly sensitive,

* sensitive,

ENO-5=Enrofloxacin (5 μg), AMC-5=Amoxicillin+Clavulanic acid (20/10 μg), T-30=Oxytetracycline (30 μg), XNL-30=Ceftiofur sodium (30 μg), GM-10=Gentamycin (10 μg), and SXT=Sulfamethoxazole trimethoprim (1.25/23.7 μg). “S” stands for sensitive, “I” for intermediate, and “R” for resistant

**Figure-4:**
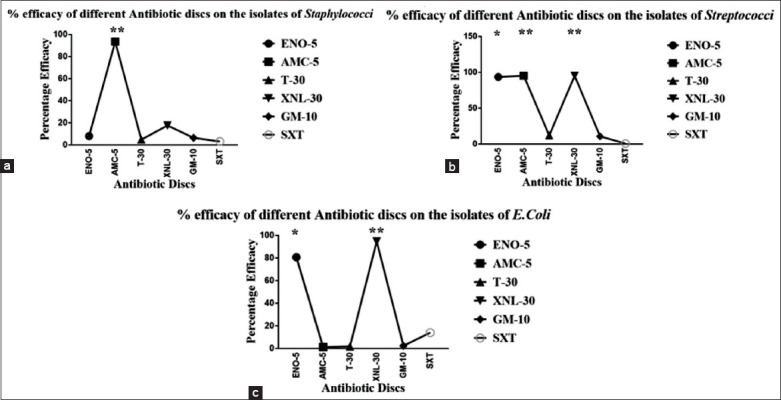
Antibiotic susceptibility profile of (a) staphylococci, (b) streptococci, and (c) *Escherichia coli*.

Amoxicillin+clavulanic acid was determined to be the drug of choice against *Staphylococcus*; amoxicillin+clavulanic acid, ceftiofur sodium, and ENO against *Streptococcus*; and ceftiofur sodium and ENO against *E. coli*. The percentage efficacy of the different antibiotics is shown in Figure-4a-c. Our findings are very close to that of Aqib *et al*. [15] and Joshi *et al*. [65], who reported that amoxicillin was the most effective and isolates of *Streptococcus* and *E. coli* have shown higher susceptibility to ceftiofur sodium and ENO, respectively, as these drugs are not regularly used for treatment in this region which might explain for their higher efficacy. However, this was a counterstatement to Ali *et al*. [21], who claimed that the efficacy of gentamicin was higher than other antibiotics against *in vitro* mastitis bacteria. It was studied that *in vitro* efficacy of tetracycline was highest (90.4%) against bacterial isolates of milk [66]. Similarly, the sensitivity of tetracycline was reported to be at 80.7% by Ali *et al*. [21] and 93.7% by Rola *et al*. [67]. Furthermore, tetracycline was identified to have the highest efficacy against mastitis bacteria found in goats [66,68,69]. Similarly, findings of Ahmed *et al*. [37], Sumathi *et al*. [70], Mir *et al*. [71], and Ceniti *et al.*, [72] are opposite to our study. There has been difficulty in comparing different studies as the criteria for interpretation and methods of resistance or susceptibility used vary [72].

### Risk factors

To improve udder health, it is necessary to determine the risk factors associated with SCM [13]. Reoccurrence of infection or the risk of infection can be attributed to factors such as poor treatment protocol during late lactation [14,15], low body score, long teats, milk fever, season, and prophylactic hygiene management; furthermore, infections in health-care centers have been identified as the major contributors to mastitis prevalence throughout the world [16-18].

This study has determined the association of several risk factors such as herd structure, deworming, vaccination, presence of ticks, use of teat dip and supplements (mineral), feeding type, age, housing, blood in the milk, milk leakage, milk taste, parity, milk yield, and ease of milking in the occurrence of mastitis in goats. These factors were analyzed statistically to determine their association (Table-1). Then, the association of risk factors and the occurrence of mastitis were analyzed using the MLR model. Initially, 14 variables that produced p<0.001 in the univariate analysis (Table-5) were included in the MLR model. However, the final model only contained 11 statistically significant variables (Table-6).

**Table-5:** Association of baseline variables with mastitis.

Variable	Group	Mastitis positive (%)	Mastitis negative (%)	χ^2^	df	p-value
Herd structure	Goats	44 (8.8)	140 (28)	177.6	3	<0.001
	Goats+Sheep	202 (41)	37 (7.4)			
	Goats+Bovine	63 (12)	14 (2.8)			
Deworming	Yes	55 (11)	254 (50.8)	10.7	1	<0.001
	No	55 (11.6)	133 (26.6)			
Vaccination	Yes	39 (7.8)	67 (13.4)	35.6	1	<0.001
	No	270 (54)	124 (24.8)			
Ticks	Yes	58 (11.6)	16 (3.2)	10.1	1	<0.001
	No	251 (50.2)	175 (35)			
Use of teat dip	Yes	6 (1.2)	42 (8.4)	54.7	1	<0.001
	No	303 (60.6)	149 (29.8)			
Supplement (mineral)	Yes	4 (0.8)	44 (8.8)	64.3	1	<0.001
	No	305 (61)	147 (29.4)			
Feeding type	Fodder	284 (56.8)	106 (21.2)	99.7	2	<0.001
	Fodder+concentrate	19 (3.8)	32 (6.4)			
	Concentrate	6 (1.2)	53 (10.6)			
Age (years)	2-3	33 (6.6)	62 (12.4)	70.6	6	<0.001
	3-4	42 (8.4)	51 (10.2)			
	4-5	45 (9)	27 (5.4)			
	5-6	48 (9.6)	19 (3.8)			
	6-7	51 (10.2)	13 (2.6)			
	7-8	46 (9.2)	10 (2)			
	>9	42 (8.4)	9 (1.8)			
Parity	2-3	87 (17.4)	75 (15)	55.4	2	<0.001
	3-4	131 (26.2)	65 (13)			
	>5	91 (18.2)	51 (10.2)			
Housing	Shed	4 (0.8)	96 (19.2)	302.0	3	<0.001
	Backyard shed	1 (0.2)	46 (9.2)			
	Tethering	85 (17)	19 (3.8)			
	Loose	219 (43.3)	30 (6)			
Blood in milk	Yes	51 (10.2)	1 (0.2)	32.3	1	<0.001
	No	258 (51.6)	190 (38)			
Milk leakage	Yes	33 (6.6)	1 (0.2)	18.5	1	<0.001
	No	277 (55.4)	190 (38)			
Milk taste	Sweet	121 (24.2)	46 (9.2)	115.4	2	<0.001
	Bitter	42 (8.4)	112 (22.4)			
	Salt	146 (29.2)	33 (6.6)			
Milk yield	Normal	196 (39.2)	180 (36)	60.1	1	<0.001
	Decreased	113 (22.6)	11 (2.2)			
Ease of milking	Positive	11 (2.2)	21 (19.2)	10.9	1	0.010
	Negative	298 (59.6)	170 (34)			

**Table-6:** Summary of key risk factors associated with the occurrence of mastitis in Beetal goats: variables included in final logistic regression model.

Characteristics	*OR	95%** CI	p-value
Herd structure			
Goats	34.4	7.2-164.2	0.00
Goats+Sheep	44.6	4.3-464.5	0.01
Goats+Bovines	1		
Housing			
Shed	1		
Backyard shed	0.002	0.03	0.00
Tethering	0.002	0-0.3	0.012
Loose	1.2	0.3-4.8	0.77
Deworming			
No	3.97	0.25-0.45	0.11
Yes	1		
Vaccine			
No	4.853	0.9-23.57	0.05
Yes	1		
Ticks			
No	0.25	0.05-1.25	0.09
Yes	1		
Feeding			
Fodder	0.48	0.07-3.09	0.44
Fodder+Concentrate	0.001	0.00-0.06	0.001
Concentrate	1		
Supplement (mineral)			
No	0.5	0.003-0.88	0.04
Yes	1		
Age			
2-3 years	0.48	0.08-2.9	0.41
3-4 years	4.6	0.52-40.11	0.17
4-5 years	3	0.44-20.23	0.27
5-6 years	2.8	0.18-45.2	0.47
6-7 years	1.4	0.09-21.7	0.8
7- 8 years	21.95	0.78-615.5	0.07
9 and above year			
Parity			
1-2 years	1		
3-4 years	0.09	0.02-0.44	0.003
More than 5	1.28	0.7-1.7	0.08
Teat dip			
No	0.14	0.02-1.01	0.05
Yes	1		
Blood in milk			
No	37.6	5.12-274.2	0.000
Yes	1		
Milk leakage			
No	49.8	1.3-1924	0.036
Yes	1		
Milk taste			
Sweet	1		
Bitter	0.61	0.15-2.45	0.48
Salt	0.18	0.4-0.85	0.3
Milk yield			
Normal	49.8	2.7-932.3	0.009
Decrease	1		
Ease of milk			
No	0.04	0.004-0.38	0.005
Yes	1		

* OR=Odds ratio;

** CI=Confidence interval

In Pakistan, it is common that milking of goats is done by hands as herds are smaller in number or are not mechanized, increasing the risk for mastitis due to poor management and hygiene practices of milk handlers [21,43,73]. In the herd, a diseased animal increases the chances of intramammary infection, which means treatment and culling policy can overcome SCM prevalence. It is reported that if culling of animals positive for CM is not done, then it will lead to the further spread of contagious mastitis in the herd [74]. Our findings on herd structure are consistent with that of Aqib *et al*. [15] while opposite to that of Megersa *et al*. [18], who stated that there is no significant difference between herd structure and occurrence of mastitis in goats. It was observed that dairy farmers do not follow the management and biosecurity standard operating procedures; for example, there is often lack of proper deworming in newborn animals [75]. It is reported that in cattle, deworming has a significant correlation with the occurrence of mastitis [76]; however, it was contrary to the findings observed by Kao *et al*. [5].

Due to vaccines against mastitis lacking widespread efficacy, new vaccine development is under way [77]. The current study revealed a strong association of vaccination of common diseases with the occurrence of mastitis due to common diseases, immune status of the animal becomes low. Therefore, occurrence of mastitis chances becomes high, while finding of Kao *et al*. [5] is opposite to the current study that stated that there is no significant difference among vaccinations against common diseases with the occurrence of mastitis in goats.

The current study showed a strong association of ticks with the occurrence of mastitis as ticks mostly infest udder causing teat and skin lesions, help in the entry of bacteria, and mark permanent damage of tissue, which may increase gradually to teats and udders. It results in a significant portion of the udder lesion and infestation of ticks was positive with mastitis. It is reported that when there was 72% infestation of ticks on the udder than mastitis occurrence. It was 30% more than non-infested udder [78]. Similarly, it was observed that the prevalence of mastitis and the infestation of ticks and lesions of the udder are correlated [79]. Thus, mastitis and other problems of the udder in animals can be reduced using acaricide to control ticks. It is suggested that during the non-lactation period of animals, ticks should be removed from the body of animals by gentle rotation and firm move without damaging the skin of the udder [80].

It is observed that animals having long teats, pendulous and deep udder, late lactation, low body condition scoring, and poor management and hygiene are more susceptible to udder infection such as mastitis and udder inflammation as compared to others [81]. Our finding on how teat dip and mastitis occurrence are correlated is similar to that in Casu *et al*. [82] and Barkema *et al*. [83]. The immunity that comes from supplements like minerals has a beneficial effect on the health of the udder [84]. Mineral supplementation is recommended throughout the year as it can enhance the immunity of dairy animals [85]. For example, selenium has been identified of having high beneficial effect on immune cell activity in cows and, rarely, in heifers [86]. Our findings of mineral supplementation are very close to that in Coe *et al*. [85], and findings related to feeding and occurrence of mastitis are similar to the findings of Aqib *et al*. [15], and Altaf *et al.*, [33], while opposite to Kao *et al*. [5].

Determination age and parity have been identified as the major factors of SCM; this as age and parity increase, occurrence of mastitis also increases due to the increase in SCC [21,87]. This was consistent with the findings of Megersa *et al*. [18], Ferdous *et al*. [47], Da Silva *et al*. [68], Piepers *et al.*, [84] who demonstrated the association of goat mastitis prevalence and gradual increase in age and parity number. Oppositely, it is reported that there is no significant association of mastitis with parity, which is opposite to our findings [5,15,33,87].

Our findings on how housing and occurrence of mastitis are associated and consistent with Kao *et al*. [5], Aqib *et al*. [15] and Kumar *et al.*, [66]. Ease of milking, milk leakage, blood in the milk, milk taste, and milk yield have been determined to have the strongest connection with the occurrence of mastitis, showing a highly significant effect (p<0.05); this as mastitis is detected, a marked hardness of teat during milking, milk leakage, blood in milk in a severe attack of mastitis, and 20% milk yield decrease are also noted. The findings on these parameters are strongly supported by Kao *et al*. [5].

## Conclusion

Our results show that SCM among Beetal goats caused by bacterial organisms is predominant in the study area, and the percentage prevalence of *Streptococcus* and *E. coli* is predominant in SCM and CM. Amoxicillin+Clavulanic acid (AMC-5) has the highest sensitivity against *Staphylococcus* isolates, while AMC-5, XNL-30, and ENO-5 have the highest sensitivity against *Streptococcus*. XML-30 and ENO-5 have the highest sensitivity against *E. coli*.

Herd structure, deworming, vaccination, presence of ticks, use of teat dip, mineral supplementation, feeding type, age, housing, blood in the milk, milk leakage, milk taste, parity, and milk yield have been determined to have a strong association with the occurrence of mastitis; meanwhile, ease of milking has a moderate association with mastitis occurrence. We hope our findings have raised awareness on the risk factors and treatment approaches against common mastitis-causing bacterial agents.

## Authors’ Contributions

MHS, IA, and AH designed the project. AJ, MI and MZI did sampling, data collection, processing, and interpretation of results. MQ and MA analyzed the data. AJ, HS, MMT, and HAN drafted the manuscript and reviewed by all. All the authors read the manuscript and approved the content.
